# Vascular amounts and dispersion of caliber-classified vessels as key parameters to quantitate 3D micro-angioarchitectures in multiple myeloma experimental tumors

**DOI:** 10.1038/s41598-018-35788-4

**Published:** 2018-11-30

**Authors:** Marco Righi, Silvia Laura Locatelli, Carmelo Carlo-Stella, Marco Presta, Arianna Giacomini

**Affiliations:** 1 CNR – Institute of Neuroscience, Milan, Italy; 2 Department of Medical Biotechnology and Translational Medicine, University of Milano, Milan, Italy; 3 Department of Oncology and Hematology, Humanitas Cancer Center, Humanitas Clinical and Research Center, Rozzano, Italy; 4 Department of Biomedical Sciences, Humanitas University, Rozzano, Italy; 5 Unit of Experimental Oncology and Immunology, Department of Molecular and Translational Medicine, University of Brescia, Brescia, Italy

## Abstract

Blood vessel micro-angioarchitecture plays a pivotal role in tumor progression, metastatic dissemination and response to therapy. Thus, methods able to quantify microvascular trees and their anomalies may allow a better comprehension of the neovascularization process and evaluation of vascular-targeted therapies in cancer. To this aim, the development of a restricted set of indexes able to describe the arrangement of a microvascular tree is eagerly required. We addressed this goal through 3D analysis of the functional microvascular network in sulfo-biotin-stained human multiple myeloma KMS-11 xenografts in NOD/SCID mice. Using image analysis, we show that amounts, spatial dispersion and spatial relationships of adjacent classes of caliber-filtered microvessels provide a near-linear graphical “fingerprint” of tumor micro-angioarchitecture. Position, slope and axial projections of this graphical outcome reflect biological features and summarize the properties of tumor micro-angioarchitecture. Notably, treatment of KMS-11 xenografts with anti-angiogenic drugs affected position and slope of the specific curves without degrading their near-linear properties. The possibility offered by this procedure to describe and quantify the 3D features of the tumor micro-angioarchitecture paves the way to the analysis of the microvascular tree in human tumor specimens at different stages of tumor progression and after pharmacologic interventions, with possible diagnostic and prognostic implications.

## Introduction

The micro-angioarchitecture of a tissue is a major parameter underlying its functional properties in terms of oxygen supply, nutrient and drug delivery. Underdeveloped or occluded vascular trees can fail in this goal^1^, but insufficient supply can also be observed in hypervascularized tumor tissues. There, uncontrolled angiogenesis, together with other mechanisms^2^, can cause abnormal vascular growth of dysfunctional vessels^3^ reducing local blood fluxes to marginal speeds and fostering hypoxic conditions^4^. Thus, the development of methods able to quantify vascular trees and their anomalies appears to be of pivotal importance for a better understanding of the neovascularization process in cancer and for the evaluation of vascular-targeted therapies. Unfortunately, it is usually hardly possible to take into consideration all the details that characterize a tissue angioarchitecture without losing the possibility to perform a concise and meaningful description.

In the past, the classical approach focalized on each single vessel idealized as a tube connecting 2 nodes (i.e. branching points) from which daughter vessels branch to reach other destinations. Based on these “objects”, the amounts, lengths, calibers, tortuosities and branching of vessels in a vascular tree could be calculated as statistical parameters and eventually correlated with specific physio-pathological states of the analyzed tissue^5–7^. With the onset of the digital revolution, the focus switched from vessels to pixels, or voxels, representing vascular tubes. In addition, this shift promoted the analysis of vascular structures as a whole, trying to keep trace of the structural and space-filling information coded into the spatial arrangement of vessels and capillaries by means of connected voxels.

In this context, a certain attention was addressed to the fractal aspects of vascularization^8^. The fractal concept provides the theoretical basis to define the fractal dimension of an object that does not completely fill the surrounding space/volume^9^. In an angioarchitecture, the fractal dimension allows to define the space-filling value of a defined vascular tree as an intermediate dimension between a flat surface (D = 2) and the volume of a solid object (D = 3)^10^ and thus promises to classify vascular trees. Unfortunately, a single value can hardly discriminate among the arrangement of microvessels with different calibers and the complex relationship and intertwisting in which they could evolve. Thus, fractal dimension is usually considered only one of the parameters concurring to the definition of the multifaceted vascular arrangements observed in neoplastic tissues.

In recent years, technological improvements have allowed the study of human tumor vasculature by different imaging techniques, including magnetic resonance angiography (MRA) (see^11^ and references therein) and acoustic angiography^12^. MRA permits the quantitative, statistical assessment of parameters such as vessel number, vessel radius, and vessel tortuosity. However, MRA image acquisition precludes the resolution of microvessels smaller than 0.5 mm in diameter. A better resolution can be achieved by acoustic angiography that can be used to define the spatial coordinates occupied by individual vessels, thus allowing the quantification of vascular tortuosity metrics for early tumor detection and evaluation of the changes in the vascular microenvironment during tumor progression^13–15^. However, also in this case, the resolution of contrast-enhanced images of superficial microvasculature (~150 μm) does not allow the analysis of capillaries or neoangiogenic sprouts.

Thus, the absence of an easily-understandable, concise, yet multifaceted picture of a microvascular status contrasts with the need of quantifying the results obtained upon pharmacological aggression of a tumor^16^ or the advantage of monitoring the progresses of vascular-targeted therapies^17^. To cope with these problems, we tried previously a different 3D approach based on the calculation of the amounts and spatial dispersion of caliber-classified tumor microvessels detected by sulfo-biotin stainining of human tumor xenografts in mice that successfully revealed specific differences following vascular-targeted therapies^18^. However, like fractal analysis, our approach failed to define a limited set of parameters useful to compare angioarchitectures by statistical analysis.

Here, in a reiterated attempt to attain this goal, we investigated the spatial relationships among the different classes of vessels of decreasing calibers composing the vascular tree. In this new approach, we considered the vascular tree as a whole rather than restricting the analysis to independent caliber-filtered classes of vessels. Image analysis of the functional microvascular network of human multiple myeloma KMS-11 xenografts in NOD/SCID mice showed that amounts, spatial dispersion and spatial relationships of adjacent classes of caliber-filtered microvessels provide a near-linear graphical “fingerprint” for a given micro-angioarchitecture. Position, slope and axial projections of this graphical outcome reflect biological features and summarize the properties of tumor micro-angioarchitecture in the absence and in the presence of vascular-targeted treatments.

## Results

### Analysis of spatial relationships among adjacent classes of caliber-filtered microvessels

**Figure 1:**
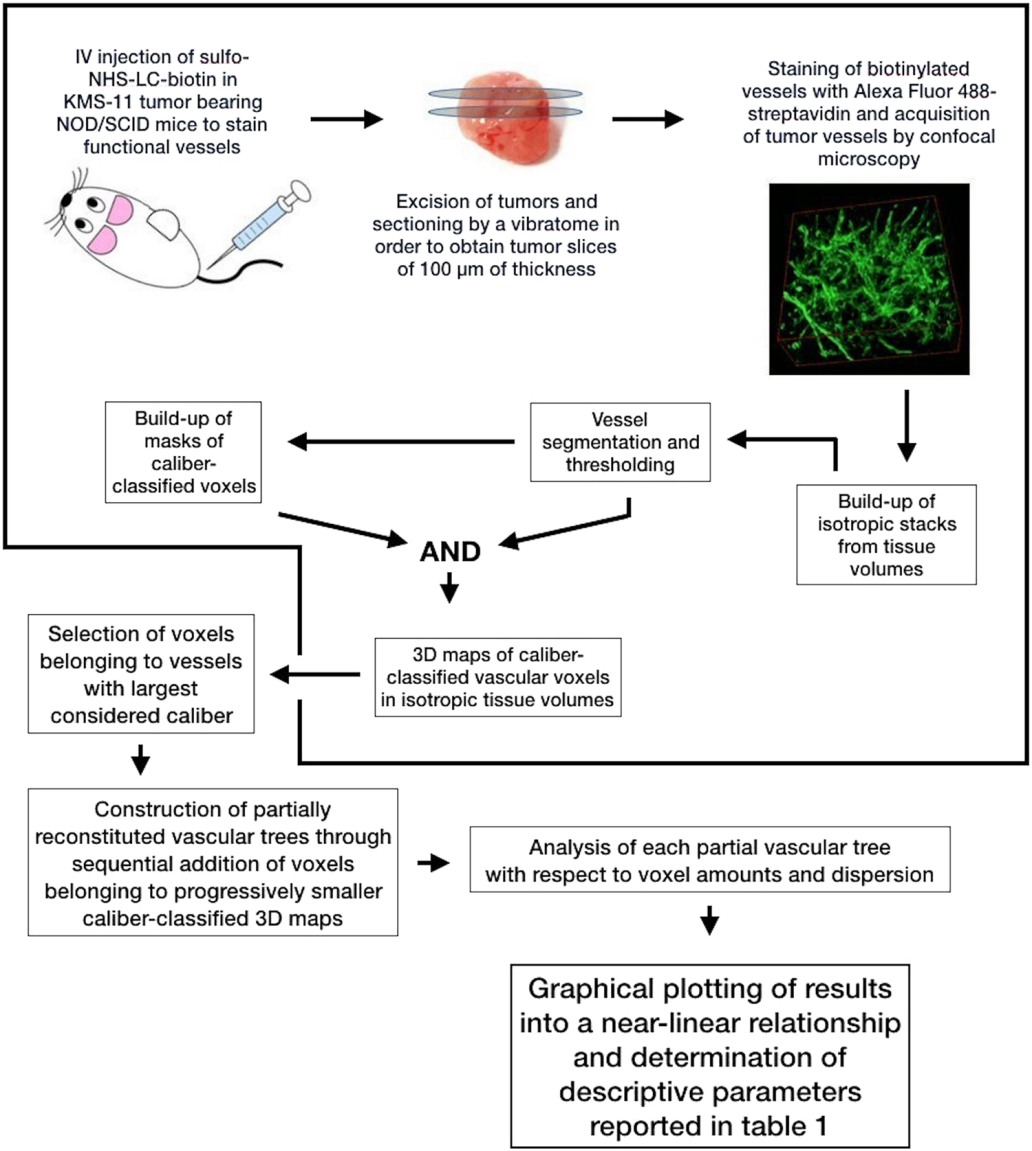
Schematic overview of the data analysis process. Steps enclosed in the bold line were previously described^18^.

**Figure 2:**
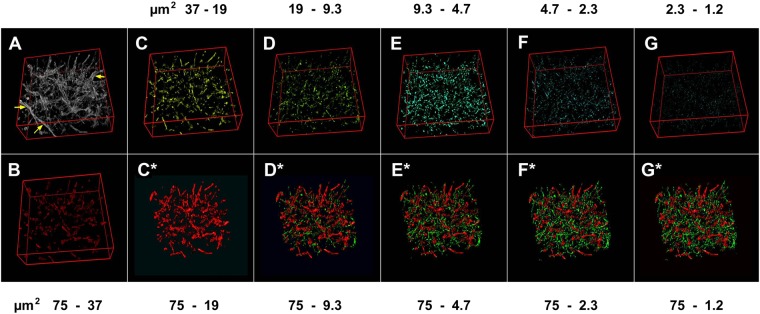
Example of cumulated microvascular images from caliber-filtered classes of identified vessels. Image A renders an 8-bit stack representing a complete vascular bed from an untreated KMS-11 tumor. Panel B is the rendering of that same image stack showing only vessels with the largest cross-section area considered (75–37 µm^2^). Panels C to G are renderings of image stacks showing vessels with cross-sections lower than 37 µm^2^ as listed across the top of the figure. Panels C* to G* are renderings of image stacks obtained after sequential, cumulated addition to B of vessels showing lower projected cross-sections classified in descending order (**C**–**G**). Thus, C* to G* images group vessels from more than a single vascular class *e*.*g*. B + C = C*, C* + D = D*. Vascular components were arbitrarily color-coded as large (75–19 µm^2^; red), medium (19–4.7 µm^2^; green), and small (4.7-1.2 µm^2^; cyan). Yellow arrows in A point to vessels with cross-section larger than 75 um^2^ and thus too big to be taken into account. Panels A to G were obtained from a previous publication^18^ and are reported to highlight the differences between the two analyses. A last couple of panels, H (1.2–0 µm^2^) and H* is not shown.

In our previous work^18^, we quantified the sulfo-biotin-stained vasculature of human multiple myeloma KMS-11 xenografts by a 3D approach based on amounts and spatial dispersion of caliber-classified microvessels taking into consideration the fluorescent signal upon subdivision of vessels according to their approximated caliber (i.e. their minimal projected cross-section area). This approach focused on the amounts and dispersion of each class of vessels but totally disregarded their relationships with the surrounding vessels with different calibers, in particular those having immediately lower calibers. In order to take into consideration this information, in the present study we re-constructed partial vascular trees of the same tumor grafts by cumulating image stacks of individual classes of vessels starting from large vessels and summing, step by step, binary signals classified by progressively lower cross-sections (Fig. 1). In order to highlight the differences between the two approaches, Fig. 2 provides an explanation of the procedure using a set of images similar to that used in our previous work^18^. Practically, we started with binary signals from vessels with the largest cross sections area (75–37 µm^2^) (Fig. 2B) and, one at a time, we added them the binary signals from the six remaining classes of smaller vessels, ordered by decreasing vascular cross-section values (Fig. 2C–G). This generates progressively cumulated vascular arrangements until obtaining the whole vascular tree (Fig. 2B–H*). Last point H* being not shown.

**Figure 3:**
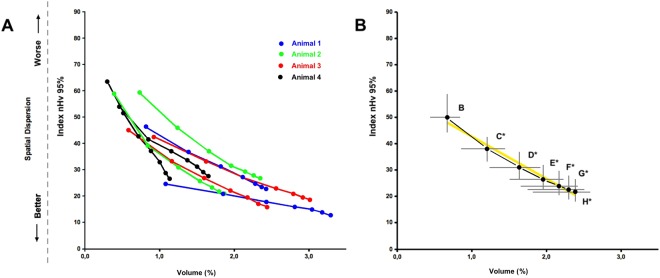
Graphical representation of vascular relationships among adjacent classes of caliber-filtered microvessels. (**A**) Eight different 3D vascular samples obtained from four KMS-11 tumor grafts (two z-stacks per tumor; one tumor per animal) were analyzed. Points representing data from increasingly reconstituted vascular trees are united by a segmented line. (**B)** Median representation of the same data: the heavy black line is the median curve obtained by calculating and plotting the median value for each point from all the samples (indicated by letters as in Fig. 2) and then tracing a segmented line. In addition to the median curve, the plot reports in gray the values of the interquartile range 25–75% (IQR) for both dimensions. The large, yellow line in the background represents the straight line fitted from median values. Both plots report on the X-axis the percent volume (V%) occupied by voxels from partially reconstituted vascular trees. Conversely, the Y-axis shows the correspondent spatial dispersion of the signal (nHv_95%_) expressed considering the normalized number of expansion cycles needed to fill 95% of the volume following a rhombicuboctahedral expansion scheme^18^.

These cumulated vascular arrangements were then analyzed for the percent amount of identified vascular signals (% vascular voxels/total voxels) as well as for signal dispersion in the volume. In order to obtain this last parameter, starting from the input vessels, we calculated the number of cycles of expansion needed to fill at least 95% of the volume (nHv_95%_ value) according to mathematical morphology rules and to a rhombicuboctahedral expansion scheme. This approach, related to the calculation of the map of euclidean distances^19^, was performed using a custom ImageJ plugin we previously developed^18^. Vascular amounts and dispersion data from each point of cumulated vascular arrangements were then plotted in a 2D space defined by the percentage of occupied volume *versus* the number of expansion cycles (V/D plot), where a lower number of cycles (i.e. a lower nHv_95%_ value) indicates a better spatial distribution of the vascular tree (Fig. 3).

### Linear relationships of cumulated microvascular arrangements

Analysis of eight different 3D vascular samples obtained from four KMS-11 tumor grafts (two z-stacks per tumor; one tumor per animal) revealed that the organization of the results relative to each sample follows an unexpected near-linear sloping line (Fig. 3A). In this sequence, the first left-end point of the line was that relative only to vessels with the largest cross-section area (75–37 µm^2^) whereas all the other points represented the data from intermediate cumulated microvascular arrangements till the last right-end point representing data obtained from the sum of all the vessels considered in the analysis (*i*.*e*. the whole vascular tree formed by vessels with cross-section area equal or lower than 75 µm^2^). For each sample, connection of all microvascular arrangements points resulted in segments very close to a straight line with a limited variability in position and slope between the two z-stacks from the same animal (Fig. 3A) and among the four tumors analyzed (Fig. S1). Accordingly, the median curve obtained from the median values of each class of partially reconstituted trees for all the eight samples examined (black dots *plus* interquartile ranges IQR in grey) confirmed the near-linear arrangement of the data with a corresponding linear regression coefficient R^2^ = 0.982 (Fig. 3B). Indeed, only 3 out of the 8 specimen curves representing KMS-11 tumor grafts showed a R^2^ value lower than 0.990, the lowest being 0.955 (Table S1), pointing to a high reproducibility of the observed near-linear organization of data from the different analyzed microvasculatures. Notably, analysis of the vascular trees obtained from three tumor grafts did not revealed any significant change in the near-linear organization of the data or in the slope of the curve after random removal of 5 or 10% of vascular signal. This indicates that artefactual vascular fragmentation (due for example to imperfect sulfo-biotin staining, bleaching of fluorescent signal, digital vessel segmentation and thresholding) does not significantly alter the analysis (Fig. S2). Altogether, these findings indicate the possibility to represent a micro-angioarchitecture with a near-linear segment that allows describing such a complex arrangement of vessels with only few parameters.

### Extrapolation of descriptive parameters from near-linear curves and their biological meanings

Descriptive parameters aimed at linking geometrical descriptors with explicit biological meanings were extrapolated from the analysis of KMS-11 tumors near-linear curves (Table 1). In particular, for each curve we calculated its slope, position of its left-end and right-end points and curve lengths after projection on the two axes of the plot (Tables 1 and S1).

**Table 1:** Biological meanings of near-linear curve-derived descriptive parameters.

Parameter	Description	Biological meaning
Slope (${\mathbb{R}}$)	shallow	Short intervascular distances
	steep	Long intervascular distances
X/Y coords of left-end point	low/low	Few large vessels/even dispersion
	low/high	Few large vessels/uneven dispersion
	high/high	Abundant large vessels/uneven dispersion
	high/low	Abundant large vessels/even dispersion
X/Y coords of right-end point	low/low	Low total vessel density/even dispersion
	low/high	Low total vessel density/uneven dispersion
	high/high	High total vessel density/uneven dispersion
	high/low	High total vessel density/even dispersion
X length	short	Decreased microvascular density
	long	Increased microvascular density
Y length	short	Limited contribution of smaller vessels to the distribution of the total tumor vasculature
	long	Significant contribution of smaller vessels to the distribution of the total tumor vasculature

In this context, the left-end point of the curves provides information about the initial subset of large vessels (cross-section area between 75 and 37 µm^2^), giving a rough description of the initial angioarchitecture supporting microvessel deployment. Thus, for a given angioarchitecture, the position of the left-end point represents both the total percent volume occupied by large vessels as well as an indication on their even or uneven distribution, with consequent effects on capillary deployment. Similarly, the right-end point of the curves provides information about volume and dispersion of the total vascular tree. Furthermore, the length of the projection of the segment on the X-axis pinpoints the percent amount of volume occupied by vessels with a cross-section ≤37 µm^2^. Conversely, the length of the projected segment on the Y-axis outlines the gain in even vascular distribution due to vessels with cross-sections ≤37 µm^2^; thus, longer segments indicate a higher importance of smaller vessels in order to obtain an even vessel dispersion. Finally, segment slope, calculated as the ratio between vascular dispersion in the volume and the amount of vascular signal (${\mathbb{R}}$), represents the intervascular distances (considering at least 95% of the tumor volume) following the “addition” of a unitary % volume of signal from vessels with decreasing calibers. It must be pointed out that the negative slope value is due to the inverse correlation between the plotted parameters. Indeed, the spatial dispersion index nHv_95%_ decreases with the increase of volume permeation, *i*.*e*. when the same vessels are better dispersed in the volume. Thus, this ratio shows the contribution of increasingly smaller vessels in reducing the intervascular distances. Consequently, for a defined angioarchitecture, a flat slope reflects a limited improvement in vessel dispersion throughout the volume in spite of considering new vessels with lower calibers and highlights an increased microvessel density adjacent to vessels with a larger caliber. Conversely, a steeper slope indicates that progressively smaller microvessels markedly improve vessels dispersion in the tissue volume considered, but at the same time reflects a micro-angioarchitecture characterized by fewer and distant neovessels. Note that vessels with lower calibers should not forcibly originate from existing larger vessels, given that they may represent infiltrating vessels generated outside the considered volume.

### Quantification of micro-angioarchitectural changes after vascular-targeted treatments

**Figure 4:**
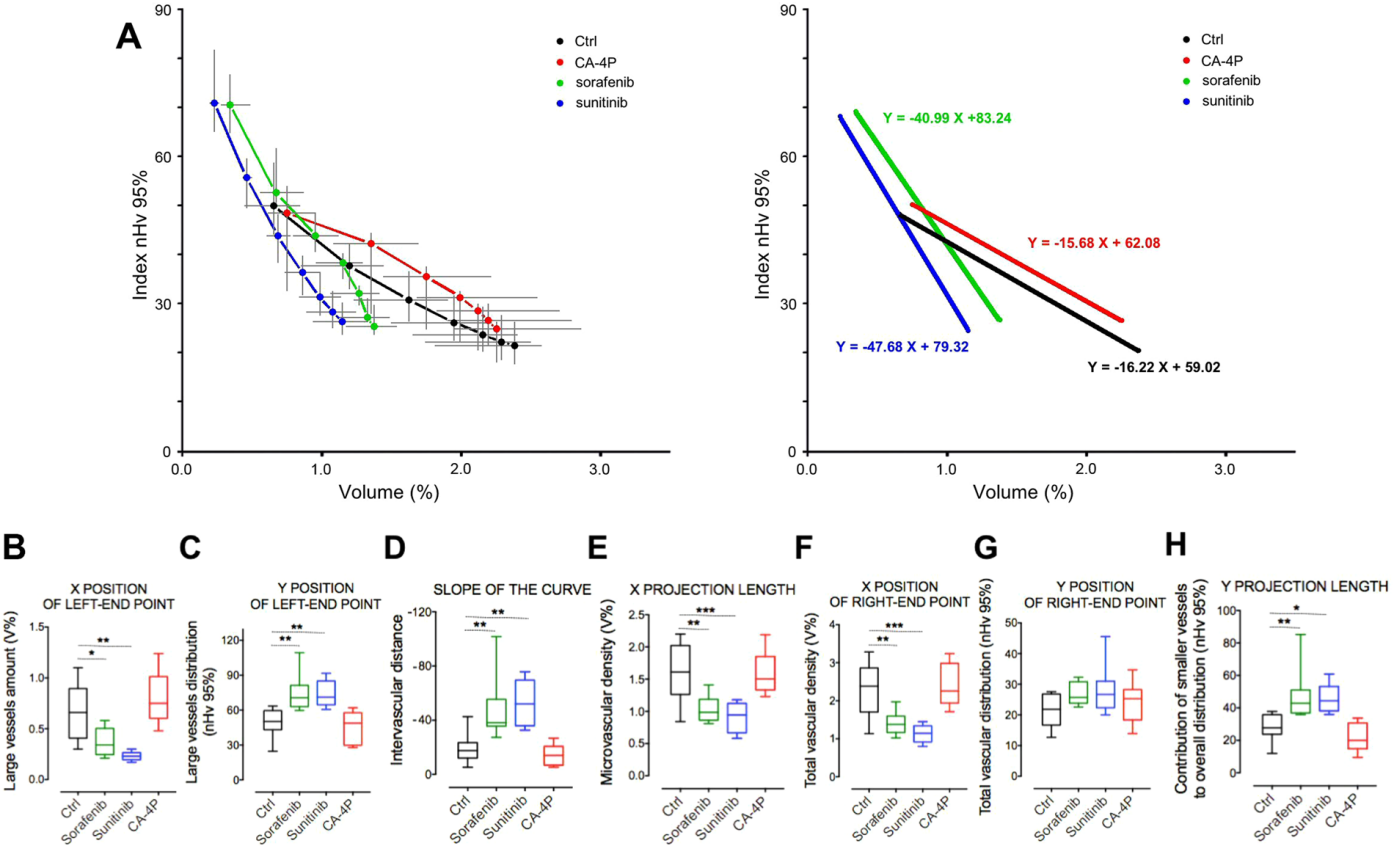
Analysis of micro-angioarchitecture after tumor treatment with vascular-targeted drugs. *Left panel*: median curves obtained from the analysis of eight 3D vascular samples obtained from four treated or untreated KMS-11 tumor grafts (two z-stacks per tumor; one tumor per animal). Median values (dots) and IQR (gray lines) for both percent volume and spatial dispersion are shown for each class of vascular trees. *Right panel*: linear regression curves calculated from the median curves shown in the left panel. (**B–H**) Box and whiskers plots of curve-derived vascular parameters. The boxes extend from the 25th to the 75th percentiles, the lines indicate the median values, and the whiskers indicate the range of values. *p < 0.05; **p < 0.01; ***p < 0.001.

To assess the capacity of our approach to reflect and quantify changes induced in tumor micro-angioarchitecture by pharmacologic approaches, we analyzed original data obtained from KMS-11 tumors treated with the two anti-angiogenic (AA) agents sorafenib and sunitinib after image re-elaboration according to the new protocol. Figure 4 and Table 2 show the median values obtained from the analysis of eight z-stacks obtained from four treated or untreated KMS-11 tumor grafts (two z-stacks per tumor; one tumor per animal). In spite of marked differences among curves, the near-linear relationship observed in untreated tumors was not significantly affected by the different pharmacologic treatments, as confirmed by the analysis of each z-stacks in which near-linear relationships were predominant, as defined by their R^2^ index values (Tables S1–S4). Indeed, upon fitting of 32 raw data series from all samples, we observed only three cases with a R^2^ index lower than 0.950, with the lowest being equal to 0.940 (Tables S2–S4). Also, a very limited variability of median curves (Fig. S1) and single data point position (Table S5) was observed among animals from the same group of treatment.

**Table 2:** Quantification of micro-vascular changes after vascular-targeted treatments.

KMS-11 tumors	Ctrl	Sorafenib	Sunitinib	CA-4P
R^2^	0.982	0.987	0.986	0.979
Slope (${\mathbb{R}}$)	−16.22 (−18.71/−13.72)	−40.99 (−46.47/−35.51)	−47.68 (−54.26/−41.11)	−15.68 (−18.36/−13.01)
X/Y coords of left-end point	0.66 (0.49)/50.20 (16.09)	0.34 (0.25)/70.68 (18.32)	0.23 (0.08)/71.04 (20.35)	0.75 (0.41)/48.78 (27.91)
X/Y coords of right-end point	2.38 (0.77)/21.82 (8.60)	1.38 (0.37)/25.71 (6.12)	1.15 (0.40)/26.70 (6.53)	2.26 (0.91)/25.29 (8.26)
X length	1.61 (0.50)	0.99 (0.27)	0.94 (0.40)	1.51 (0.48)
Y length	27.73 (9.84)	40.04 (9.35)	40.43 (9.69)	20.33 (10.57)

Data are from the analysis of eight 3D vascular samples (z-stacks) obtained from four KMS-11 tumor grafts per group of treatment (two z-stacks per tumor; one tumor per animal). For each type of treatment, median vascular amounts and dispersion data from each point of cumulated vascular arrangements were used to obtain an interpolating line by linear fitting. Fitted lines were characterized in terms of R^2^, slope (${\mathbb{R}}$), X/Y coordinates of the first (left-end) and the final (right-end) points and the X/Y length after projection on the respective axes together with their relative IQR inside brackets.

Administration of the AA agents sorafenib and sunitinib caused large alterations of the KMS-11 tumor angioarchitecture (Fig. 4 and Tables S1–S3). Both treatments resulted in average V/D plots showing an upward and leftward shift of the initial left-end point (Fig. 4A), indicating a marked loss in vessels with a cross-section between 75 and 37 µm^2^ (Fig. 4B) and a drop in their even distribution (Fig. 4C). In addition, the data points appeared organized in a steeper linear relationship (Fig. 4A) indicating an increase in intervascular distances (Fig. 4D). As a result, the length of the X-projection of these curves was markedly shortened, indicating a significant reduction in the amount of all vessels down to the smallest ones (Fig. 4E). Accordingly, a leftward shift of the final right-end point was observed (Fig. 4A), indicating a marked reduction of total vascular density (Fig. 4F). As to vascular distribution, all data points appeared shifted to higher nHv_95%_ values, pointing to an increased dispersion of all classes of treated vessels when compared to controls (Fig. 4A). This occurred despite the higher contribution of smaller vessels to the distribution of the total tumor vasculature, as indicated by the absence of a significant upward shift of the right-end point (Fig. 4A,G) paralleled by increased Y-projections length following AA drug treatment (Fig. 4H). This may reflect the “pruning” activity exerted by AA drugs on the tumor vasculature that leads to “normalization” of the vascular tree^20,21^.

In order to understand whether the micro-angioarchitectural changes observed with sunitinib and sorafenib were related to their anti-angiogenic mechanism of action, we extended our analysis to KMS-11 tumors treated with the vascular disrupting agent CA-4P^22^. Vascular disrupting agents exert their anti-vascular activity by shutting down established tumor vessels and inducing tumor hemorrhagic necrosis downstream the damaged vessels^23,24^. Thus, at variance with anti-angiogenic treatments that inhibit new vessels formation, a short-term CA-4P treatment is not expected to perturb significantly the tumor micro-angioarchitecture. Accordingly, analysis of CA-4P-treated tumors revealed a V/D curve very close to that of controls (Fig. 4, Tables 2 and S4).

To further assess the power of our 3D analysis to discriminate treatment-specific microvascular changes, we applied it to eight different 3D vascular samples obtained from two CD34−TRAIL^+^ cells-treated KMS-11 tumor grafts (Fig. S3, Table S6). In a previous study, using the same KMS-11 tumor model, we have demonstrated that human CD34^+^ cells engineered to express membrane-bound TRAIL (CD34−TRAIL^+^ cells) target not only tumor cells, but also tumor vasculature^25^. Indeed, CD34−TRAIL^+^ cells treatment induces apoptosis of TRAIL-R2 expressing tumor endothelial cells causing a significant reduction of vascular density and perturbing tumor vascular network^25,26^. Preliminary data obtained by our novel 3D vascular analysis showed a substantial linearity for V/D plots (Fig. S3A), with a R^2^ index equal to 0.994 for the fitted median curve (Table S6). The representative CD34−TRAIL^+^ median curve positioned the new treatment halfway between AA drug-treated and control KMS-11 tumors (Fig. S3A). Analysis of V/D plots did not show any significant shift of the initial left-end point (Fig. S3A), indicating CD34−TRAIL^+^ cells treatment did not perturb vessels with a cross-section between 75 and 37 µm^2^ (Fig. S3B,C). In contrast, a significant increase in the steepness of the slope was observed after treatment with CD34−TRAIL^+^ cells (Fig. S3A) indicating increased intervascular distance compared to controls (Fig. S3D). Accordingly, a significant reduction in the projection of the data interval on the X-axis and a leftward shift of the right-end point were observed (Fig. S3A), pointing to a reduced total microvessel density after CD34−TRAIL^+^ cell treatment (Fig. S3E,F). At variance with AA treatments, CD34−TRAIL^+^ cells did not significantly modify signal dispersion, producing only a slight upward shift of the right-end point (Fig. S3A,G) as well as a slight increase in the Y-projection of the data interval (Fig. S3H). These data show a clear effect of CD34−TRAIL^+^ cells treatment on the amount of smallest tumor vessels, distinct from that exerted by AA drug treatments on tumor angioarchitecture. Altogether, these findings suggest that our analysis may allow to discriminate efficiently among different treatment-specific micro-vascular changes.

## Discussion

The possibility to describe the complex spatial evolution of an angioarchitecture with only few parameters may represent an important point in the comparison of vascular changes among different physio-pathological states of a given tissue, including solid tumors. Unfortunately, the efforts to reach this goal have been so far frustrated by the difficulties in recapitulating the 3D arrangement of blood vessels, also because of the overwhelming amount of details that could be taken into consideration.

Up to now the angioarchitecture of a tissue is usually inferred after consideration of basic morphological parameters of the digitalized vessels, segmented after an object recognition step^27^ followed, or not, by vessel skeletonization^28,29^. Following these approaches, it is possible to calculate the microvascular density (MVD) of a tissue^5^, the lengths and branches of recognized vessels^30^, together with their caliber and tortuousity^27,31^ as well as their orientations, if 3D information are available. All these parameters concur to define the analyzed architecture but are difficult to be considered as a whole. Similarly, it is difficult to appreciate the meaning of their co-ordinated changes even when comparing related micro-angioarchitectures.

Various authors have considered unifying criteria to classify micro-angioarchitectures both at 2D or 3D level. For instance, the calculus of the fractal dimension^9^ has been extensively applied to this purpose^10,32^. More elaborated applications are those based on multifractal analysis^33–35^ which could pinpoint the differences between normal or altered vascular trees in pathologic retina or cerebral tissues. However, fractal analyses do not help to unravel the spatial relationships between vessels of close calibers, nor drive the analyst to presume the state of their connections.

Imaging techniques like MRA and acoustic angiography have allowed a quantitative evaluation of the angioarchitecture of experimental and human tumors that, however, is limited to vessels ≥150 μm in diameter. Thus, even though these techniques may provide important information about the tumor vasculature during progression of the disease or therapy, they do not allow the analysis of the architecture of tumor microvessels^11–15^.

In this paper we report the observation that the angioarchitecture of tumor vascular trees involving microvessels with an approximate cross-section lower than 75 µm^2^ detected by sulfo-biotin staining of human multiple myeloma KMS-11 tumor xenografts in mice can be summarized in near-straight segments by plotting an elaboration of their percent amount of vascular signals *versus* their 3D dispersion. This novel approach originated a graphical “signature”, or fingerprint, of the micro-angioarchitecture under analysis. Signal voxels were classified as belonging to vessels of a defined caliber and were analyzed according to this parameter, taking into account their spatial relationships with cognate signals from vessels with smaller calibers. At variance with our previous approach^18^, now we took into consideration the amounts and dispersions of progressively cumulated groups of vessels characterized by decreasing cross-sections rather than restricting the analysis to individual classes of vessels described independently from each other.

Unexpectedly, we found that the ratio between vascular dispersion in the volume and the amount of vascular signal (${\mathbb{R}}$) in multiple myeloma KMS-11 tumor xenografts was approximately constant when considering functional vessels with progressively lower calibers. This allowed the graphical representation of the micro-angioarchitecture of the tumor lesion as a straight line characterized by its position in the quadrant of the Cartesian plane, slope and projections on the X and Y-axes. Notably, these parameters varied as a function of the experimental therapeutic approaches herewith described and characterized by specific modifications of the tumor microvascular tree. As a further point, our approach had the advantage to convey precise quantitative indications on tumor angioarchitecture. In particular, the slope of the representative segment can be thought as a projection of how many vessels of immediately lower caliber were present in the tumor sample and their proximity to those with higher caliber. A steeper slope would reflect a micro-angioarchitecture characterized by fewer and distant neovessels, whereas a shallow slope would reflect the presence of a denser distribution of microvessels adjacent to those with a larger caliber. Thus, the slope of the near-linear relationship between vascular dispersion in the volume and the amount of vascular signal (parameter ${\mathbb{R}}$) can be thought as indicative of the angiogenic status of the vascular tree, with steeper segments representing poorer angiogenic trees.

To assess the capacity of our approach to describe quantitatively the changes induced in tumor micro-angioarchitecture by pharmacologic approaches, we analyzed KMS-11 tumor grafts treated with the two AA agents sorafenib and sunitinib^18^. As stated above, drug treatment did not affect significantly the linearity of the relationship between vascular dispersion in the volume and the amount of vascular signal. However, sorafenib and sunitinib exerted a different impact on the parameters describing such relationship, in line with previous observations about their impact on tumor vascularization^36–38^. In fact, analysis of the V/D plots indicates that administration of the two AA agents causes significant alterations of KMS-11 tumour vascularization^39^. Notably, the analysis showed a limited inter- and intra-tumor variability when applied on untreated as well as AA drug-treated lesions.

In keeping with our observations, recent experiments performed on human renal cell carcinoma xenografts in mice have shown that ultrasound vascular imaging can detect early alterations of tumor vasculature following treatment with sunitinib *plus* Notch inhibition with a resolution of approximately 500 μm^40^. Together, these data indicate the possibility that different, complementary approaches can be used to assess the early effects of anti-angiogenic therapies on both tumor macro and micro-vasculature.

To further assess the capacity of our model to depict changes in tumor vascularization, we investigated the micro-angioarchitectural modifications occurring in KMS-11 lesions following the administration of CD34^+^ cells engineered to express membrane-bound TRAIL (CD34−TRAIL^+^ cells)^25^. This treatment exerts proapoptotic effects both in tumor cells and tumor endothelial cells^25,26^. To this respect, a preliminary analysis of the vascular trees performed on a limited number of tumor grafts showed a clear effect of a short-term CD34−TRAIL^+^ cells treatment on the amount of the smallest tumor vessels, distinct from that exerted by AA drug treatments on tumor angioarchitecture, thus suggesting a specific action of these cytotoxic cells on the deployment of tumor vessels^41^.

The possibility to quantify the characteristics of a micro-angioarchitecture opens the way to the quantitative analysis of the pharmacologic effects of anti-tumoral agents on tumor vascular trees. From a translational point of view, this type of analysis might be performed on vascular marker-immunostained human tumor specimens to monitor micro-angioarchitectural changes during adjuvant or neoadjuvant anti-tumor therapies and, possibly, to correlate micro-vascular modifications with therapeutic outcome and prognosis. In addition, this approach might be applied in different pathological conditions in which the tissue microvasculature, and its response to therapy, may play an important role. To this regard, a preliminary analysis performed on a genetic murine model of the human neurodegenerative Krabbe disease^42,43^ has highlighted significant differences in the micro-angioarchitecture of the brain cortex of affected mice when compared to control animals (M. Righi, unpublished observation), confirming the capability of this analytical approach to discriminate normal from pathological tissues.

In conclusion, the procedure herewith described demonstrates the possibility to analyze and quantify the 3D features of the micro-angioarchitecture of human tumor xenografts in mice. Further studies are required to assess whether this approach will allow the analysis of the microvascular tree in human tumor specimens at different stages of tumor progression or after therapeutic interventions. The possibility to describe the complex arrangement of tissue microvessels with few quantitative parameters might have significant diagnostic and prognostic implications in cancer as well as in physio-pathological settings other than tumors.

## Materials and Methods

### Ethics Statement and animal treatments

The animal experiments were performed according to EU 86/109 Directive (D.L. 116/92 and following additions) and were approved by the institutional Ethical Committee for Animal Experimentation of the Humanitas Clinical and Research Center. Mice were housed under standard laboratory conditions according to our institutional guidelines.

In compliance with the 3-Rs recommendation^44^, most of the data utilized in this work originate from previously acquired images from multiple myeloma KMS-11 xenografts in control or treated mice^18^ without the use of new animals. Briefly, human multiple myeloma KMS-11 cells (5 × 10^6^ cells/mouse) were inoculated subcutaneously in the left flank of each mouse. When tumors were palpable (usually 10–12 days after tumor inoculation), they showed a diameter of 7–10 mm with a weight of 0.210 ± 0.070 g on average. Then mice were randomly assigned to receive a 5-day course of either intraperitoneal (IP) sorafenib (90 mg/kg/day) or oral sunitinib (40 mg/kg/day) or were subjected to a single IP injection of 50 mg/kg combretastatin-A4-phosphate (CA4P) 72 hours before euthanasia. In these conditions, vessels were already formed and could be targeted by the anti-vascular agent. At the same time, tumors were still growing, and tumor angiogenesis was still an active process that could be inhibited by anti-angiogenic agents.

When a new analysis was performed on KMS-11 xenografts in CD34−TRAIL^+^ cell-treated mice, tumors were obtained as above described and mice received PBS or one intravenous injection of CD34−TRAIL^**+**^ cells (3 × 10^6^ cells/mouse) 72 hours before euthanasia.

Just before sacrifice, animals were injected with sulfo-biotin^25^ and biotinylated tumors were excised and processed for microscopy analysis as previously described^18^.

### Vascular images and vessel classification by projected cross-sections

For each experimental condition, we analyzed eight different 3D vascular samples (z-stacks) obtained from four KMS-11 tumor grafts (two z-stacks per tumor; one tumor per animal). Images from 100 μm thick tumor sections were acquired as z-series at 1.0 µm distance using a confocal microscope with a 40x oil immersion objective. Then, images were pre-processed and transformed to binary vascular stacks as described^18^. Binary isotropic stacks were filtered and hollow vessels filled as already reported in order to classify input binary voxels in different classes of calibers. This step was performed according to the minimum dimension of the cross-sections passing through each voxel along the three Cartesian planes following the procedure and using ImageJ scripts as described^18^.

### Construction of progressively reconstituted vascular trees according to projected vascular cross-sections

The availability of collections of binary stacks classified according to decreasing projected vascular cross-sections was a prerequisite in order to obtain a set of progressively reconstituted vascular trees. For each sample, the initial seed stack from larger vessels (with approximated 75–37 µm^2^ cross-sections) was combined with its immediate lower neighbor in terms of vascular calibers, to obtain the sum of signal voxels (black voxels) for two close classes of vessels. Then, this new stack was fused with a 3rd carrying information from the next lower class of caliber-filtered vessels and the procedure reiterated until we obtained a set of 7 partially reconstituted vascular trees carrying information from 1 to 7 stacks down to the smallest vessels. This approach produced partially reconstituted vascular trees that, given the dimensions of the isotropic voxel (0.54 µm), grouped vessels with projected cross-sections from 74.6 µm^2^ down to 37.3, 18.6, 9.33, 4.67, 2.33, 1.17 and 0 µm^2^. Given the large number of samples involved, we wrote an ImageJ script (see Supplementary Material Script S1) to automate the process and to obtain the sets of partially reconstituted vascular trees described in the present work.

### Analysis of percent volume and spatial distribution of the reconstituted vascular trees

The percent vascular volume of a sample (V%) was calculated as the percent ratio of all black voxels to the total voxels of the volume, obtaining an adimensional value as in Righi *et al*.^18^. Similarly, we used the same ImageJ plugin described in^18^ to calculate the normalized Halo Index (nHv_95%_) as a measure of euclidean distances (spatial dispersion), according to our published rhombicubocthaedral dilation protocol. In this last case, we expressed the result obtained in cycles, *i*.*e*. the normalized number of expansion cycles needed to fill up 95% of the volume. Normalization was carried out calculating, for each sample, the number of initial cycles needed to reach the greatest initial volume observed among all the volumes to be analyzed. This sample-specific value was then subtracted from the raw total number of cycles, thus obtaining the normalized index.

To group results from samples subjected to a similar treatment, we preferred to express the data as median values rather than mean values because of their intrinsic robustness as estimators and because our spatial dispersion values are not necessarily additive. Consequently, we used interquartile range 25–75% (IQR) for both V% and nHv_95%_ values to summarize data from each group of 8 partially reconstituted vascular trees. These results were then plotted in a 2-D Volume/Spatial Dispersion plot (V/D plot) and connected together to obtain the median curve for that treatment. The median curves were fitted with straight lines and characterized in terms of slope, XY coordinates of their first left-end point, and lengths upon projection on the X- and Y- axes. Slope was represented using the parameter ${\mathbb{R}}$ defined as the ratio between vascular dispersion in the volume (nHv_95%_) and the amount of vascular signal (V%). Projected lengths of median curves were obtained by evaluating the median value of projected sample curves.

### Software and statistical analyses

All image analyses were performed using the NIH program ImageJ v. 1.48 v in the 64 bit version run on an Apple MacPro computer equipped with a 2.8 GHz Quad-Core Intel Xeon processor. Most of the macro and plugin used in this study were already published, and are available at 10.5061/dryad.6c44q. The script used to build cumulated sections is detailed as Script S1 whereas random depletion of the vascular signal was obtained by applying a custom routine as detailed in Script S2 in the supplementary material section.

Statistical analyses were performed using the statistical package Prism 7 (GraphPad Software) run on a Macintosh Pro personal computer (Apple Computers). Student’s t test for unpaired data (2-tailed) was used to test the probability of significant differences between two groups of samples. For more than two groups of samples, data were statistically analyzed with a 1-way analysis of variance, and individual group comparisons were evaluated by the Bonferroni multiple comparison test. Differences were considered significant when p < 0.05.

Similarly, data series or median curves were fitted using the non-linear fitting tool of the same program choosing straight line as the model of fitting. With this tool, we calculated ${\mathbb{R}}$ (slope) and 95% confidence intervals (CI) of slope as well as R^2^.

## Electronic supplementary material

Supplementary information

## Data Availability

The raw image datasets generated during and/or analyzed during the current study are not publicly available due to their huge dimensions but are available from the corresponding author on reasonable request. The text of the ImageJ macros used to process and combine images of tumor vessels to partially reconstituted vascular trees and for the random depletion of the vascular signal is enclosed as Supplementary Information. Previous ImageJ macros needed to process raw vascular images to binary stacks of caliber-filtered vessels can be found at the Dryad repository: ‘10.5061/dryad.6c44q’.

## References

[1] Coull BM, Clark WM (2002). Abnormalities of hemostasis in ischemic stroke. Med. Clin. North. Am..

[2] Carmeliet P, Jain RK (2011). Molecular mechanisms and clinical applications of angiogenesis. Nature.

[3] Nagy JA, Chang SH, Shih SC, Dvorak AM, Dvorak HF (2010). Heterogeneity of the tumor vasculature. Semin. Thromb. Hemost..

[4] Muz B, de la Puente P, Azab F, Azab AK (2015). The role of hypoxia in cancer progression, angiogenesis, metastasis, and resistance to therapy. Hypoxia.

[5] Weidner N (1992). Tumor angiogenesis: a new significant and independent prognostic indicator in early-stage breast carcinoma. J. Natl. Cancer. Inst..

[6] Hathout L, Do HM (2012). Vascular tortuosity: a mathematical modeling perspective. J. Physiol. Sci..

[7] Kocin´ski M, Klepaczko A, Materka A, Chekenya M, Lundervold A (2011). 3D image texture analysis of symulated and real-world vascular trees. Comput. Methods Programs Biomed..

[8] Mandelbrot, B. B. *The Fractal Geometry of Nature*. (Freeman, 1983).

[9] Glenny RW, Robertson T, Yamashiro S, Bassingthwaighte JB (1991). Applications of fractal analysis to physiology. J. Appl. Phys..

[10] Di Ieva A, Grizzi F, Sherif C, Matula C, Tschabitscher M (2011). Angioarchitectural heterogeneity in human glioblastoma multiforme: a fractal-based histopathological assessment. Microvasc. Res..

[11] Bullitt E (2009). Computerized assessment of vessel morphological changes during treatment of glioblastoma multiforme: report of a case imaged serially by MRA over four years. Neuroimage.

[12] Gessner RC, Frederick CB, Foster FS, Dayton PA (2013). Acoustic angiography: a new imaging modality for assessing microvasculature architecture. Int. J. Biomed. Imaging..

[13] Gessner Ryan C., Aylward Stephen R., Dayton Paul A. (2012). Mapping Microvasculature with Acoustic Angiography Yields Quantifiable Differences between Healthy and Tumor-bearing Tissue Volumes in a Rodent Model. Radiology.

[14] Shelton SE (2015). Quantification of Microvascular Tortuosity during Tumor Evolution Using Acoustic Angiography. Ultrasound Med. Biol..

[15] Rao SR, Shelton SE, Dayton PA (2016). The “Fingerprint” of Cancer Extends Beyond Solid Tumor Boundaries: Assessment With a Novel Ultrasound Imaging Approach. IEEE Trans. Biomed. Eng..

[16] Zhang C (2014). Comparison of dynamic contrast-enhanced MR, ultrasound and optical imaging modalities to evaluate the antiangiogenic effect of PF-03084014 and sunitinib. Cancer Med..

[17] Laufer S (2014). Monitoring brain tumor vascular heamodynamic following anti-angiogenic therapy with advanced magnetic resonance imaging in mice. PLoS ONE.

[18] Righi M, Giacomini A, Cleris L, Carlo-Stella C (2013). (3)D [corrected] quantification of tumor vasculature in lymphoma xenografts in NOD/SCID mice allows to detect differences among vascular-targeted therapies. PLoS ONE.

[19] Ragnelmam I (1993). The euclidean distance transformation in arbitrary dimensions. Pattern Recogn. Lett..

[20] Goel S, Wong AH, Jain RK (2012). Vascular normalization as a therapeutic strategy for malignant and nonmalignant disease. Cold. Spring. Harb. Perspect. Med..

[21] Jain RK (2005). Normalization of tumor vasculature: an emerging concept in antiangiogenic therapy. Science.

[22] Dark GG (1997). Combretastatin A-4, an agent that displays potent and selective toxicity toward tumor vasculature. Cancer Res..

[23] Thorpe PE (2004). Vascular targeting agents as cancer therapeutics. Clin. Canc. Res..

[24] Siemann DW (2005). Differentiation and definition of vascular-targeted therapies. Clin. Canc. Res..

[25] Lavazza C (2010). Human CD34+cells engineered to express membrane-bound tumor necrosis factor-related apoptosis-inducing ligand target both tumor cells and tumor vasculature. Blood.

[26] Giacomini A (2013). Induction of death receptor 5 expression in tumor vasculature by perifosine restores the vascular disruption activity of TRAIL-expressing CD34(+) cells. Angiogenesis.

[27] Helmberger M (2014). Quantification of tortuosity and fractal dimension of the lung vessels in pulmonary hypertension patients. PLoS ONE.

[28] Cornea ND, Silver D, Min P (2007). Curve-skeleton properties, applications, and algorithms. IEEE Trans. Vis. Comput. Graph..

[29] Tan H (2016). A robust method for high-precision quantification of the complex three-dimensional vasculatures acquired by X-ray microtomography. J. Synchrotron. Rad..

[30] Ehling J (2014). Micro-CT imaging of tumor angiogenesis quantitative measures describing micromorphology and vascularization. Am. J. Pathol..

[31] Scott A, Powner MB, Fruttiger M (2014). Quantification of vascular tortuosity as an early outcome measure in oxygen induced retinopathy (OIR). Exp. Eye. Res..

[32] Di Ieva A (2007). Fractal dimension as a quantitator of the microvasculature of normal and adenomatous pituitary tissue. J. Anat..

[33] Stošić T, Stošić BD (2006). Multifractal analysis of human retinal vessels. IEEE Transactions on Medical Imaging.

[34] Lee J, Zee BCY, Li Q (2013). Detection of neovascularization based on fractal and texture analysis with interaction effects in diabetic retinopathy. PLoS ONE.

[35] Ding Y (2015). Retinal vasculature classification using novel multifractal features. Phys. Med. Biol..

[36] Tozer GM, Kanthou C, Baguley BC (2005). Disrupting tumour blood vessels. Nat. Rev. Cancer.

[37] Wilhelm S (2006). Discovery and development of sorafenib: a multikinase inhibitor for treating cancer. Nat. Rev. Drug Discov..

[38] Wilhelm SM (2008). Preclinical overview of sorafenib, a multikinase inhibitor that targets both Raf and VEGF and PDGF receptor tyrosine kinase signaling. Mol. Cancer Ther..

[39] Gridelli C (2007). Sorafenib and sunitinib in the treatment of advanced non-small cell lung cancer. Oncologist.

[40] Rojas, J. D. *et al*. Ultrasound Measurement of Vascular Density to Evaluate Response to Anti-angiogenic Therapy in Renal Cell Carcinoma. *IEEE Trans*. *Biomed*. *Eng*., 10.1109/TBME.2018.2860932 (2018). 10.1109/TBME.2018.2860932 PMC6691901 30059292

[41] Di Bartolo BA (2015). Tumor necrosis factor–related apoptosis-inducing ligand (TRAIL) promotes angiogenesis and ischemia-induced neovascularization via NADPH oxidase 4 (NOX4) and nitric oxide–dependent mechanisms. J. Am. Heart. Assoc..

[42] Belleri M (2013). Inhibition of angiogenesis by β-galactosylceramidase deficiency in globoid cell leukodystrophy. Brain..

[43] Giacomini A (2015). Brain angioarchitecture and intussusceptive microvascular growth in a murine model of Krabbe disease. Angiogenesis.

[44] Flecknell P (2002). Replacement, reduction and refinement. ALTEX.

