# Supplementary material for: Vascular amounts and dispersion of caliber-classified vessels as key parameters to quantitate 3D micro-angioarchitectures in multiple myeloma experimental tumors
Source: Sci Rep. 2018 Nov 30;8:17520. doi: 10.1038/s41598-018-35788-4 (PMC6269464; doi:10.1038/s41598-018-35788-4)
Supplement: Supplementary file 1 — Supplementary information [file 41598_2018_35788_MOESM1_ESM.pdf]

SUPPLEMENTARY INFORMATION

Vascular amounts and dispersion of caliber-classified vessels as key parameters to quantitate 3D micro-angioarchitectures in multiple myeloma experimental tumors

Righi Marco, Locatelli Silvia Laura, Carlo-Stella Carmelo, Presta Marco, Giacomini Arianna.

Script S1. Text of the ImageJ script used to build cumulated reconstituted trees.

```
/*
ImageJ script for constructing partial angioarchitectures by cumulating sequential classes of vessels with decreasing calibers.

v. 0.1 released 29 AUG 2015

This script sums passband stacks which SHOULD be organized in a 2-level nested folder. Each subfolder will be related to a specific sample and contain caliber-classified stacks of exactly the same dimensions named in order to be listed from lower to higher calibers (low calibers first).
*/

macro "BuildingScript" {accumula();}

function accumula() {
		requires("1.42l");

// Setting time and date of execution
		getDateAndTime(year, month, week, day, hour, minu, sec, msec);
		mese = month+1;
		setBatchMode(true);

// Choosing folder grouping folders with caliber-classified image stacks ready to be combined.
		dir1 = getDirectory("Select folder grouping folders with stacks to be combined");
		list = getFileList(dir1);

// Choosing the folder for saving combined images.
		dir2 = getDirectory("Where should I save combined images?");

// Start of cycle on all nested folders
		for (st=0; st<list.length; st++) {
				subdir = dir1+list[st];
				subdirtit = File.getName(subdir);

// Creation of folder to group partially reconstituted trees
				File.makeDirectory(dir2+"SC"+subdirtit);

// Now we begin the cycle through every stack of all nested folders
				sublist = getFileList(subdir);
				titre = newArray(2);
				fulltree ="0-256";
				topdown = newArray("4-256","8-256","16-256","32-256","64-256","128-256");

// We create a working stack and for that we open the first stack of caliber-classified vessels
				open(subdir+sublist[0]);
```

```
// we want to be sure it is void, so we clean it
			run("Select All");
			run("Clear", "stack");

// we rename it - it will be the working stack
			rename("void");
			titre[0] = getTitle();

// then, for all stacks of the first subfolder - the first stack among them -
			for (i=0; i<sublist.length; i++) {

// First step: we cumulate images of caliber-classified vessels summing them to the working stack - the void image - until it will group ALL classes of caliber-classified vessels (full image)
				open(subdir+sublist[i]);
				titre[1] = getTitle();
				imageCalculator("Add stack", titre[0], titre[1]);
				selectWindow(titre[1]);
				close();
			}

// Now we save the full image of the vascular tree grouping all caliber-classified vessels
			selectWindow(titre[0]);
			save(dir2+"SC"+subdirtit+File.separator+"TD_"+fulltree+".tif");
			titre[0] = getTitle();

// Second step: we create and save partially reconstituted vascular trees (Top-down images) repetitively subtracting classes of caliber-classified vessels from the working image - the once full image - which is saved after every step, until it shows only the largest class of caliber classified vessels.
			for (i=0; i<sublist.length-1; i++) {
				open(subdir+sublist[i]);
				titre[1] = getTitle();
				imageCalculator("Subtract stack", titre[0], titre[1]);
				selectWindow(titre[0]);
				save(dir2+"SC"+subdirtit+File.separator+"TD_"+topdown[i]+".tif");
				selectWindow(titre[1]);
				close();
			}

// Now we close the working image.
			selectWindow(titre[0]);
			close();
		}

// and we stop the script
		setBatchMode(false);
		showMessage("End of Elaboration");
}
```

Script S2. Text of the ImageJ script used to randomly remove a defined portion of signal from a given binary image stack.

```
/*
ImageJ script to randomly remove a defined portion of signal from a binary stack.
The user can choose among 2 thresholds, namely 5% and 10%. The user can also decide the dimensions of a cube in which the signal is "clusterized". Actually, this parameter is limited to only 2 dimensions: 1x1x1 (single voxel) or 4x4x4 (64 voxels volume). If the user chooses this last dimension, the macro will remove all the signal found inside the chosen proportion of the 4x4x4 cubes in which the original volume could be divided.

v. 0.1 released  23 August 2018
v. 0.2 released 04 September 2018

In the case of single voxel, the script checks every voxel in the stack and, in the occurrence of a signal voxel, asks the system for a random value. If the value is higher than the threshold chosen, the signal voxel is copied to a target stack; otherwise it is discarded.
in the case of a 64 voxels volume, the script builds a 3D mask lacking up to the desired percent of 64 voxels volumes chosen randomly. The mask is then used to intersect the original binary stack, producing the final image.
The resulting image is saved in a folder chosen by the user at the onset of the elaboration.
*/

macro "Remove_signal"{eatVx();}

function eatVx(){
	requires("1.48l");

// Setting parameters through Dialog
	sval=newArray("5%","10%");
	bloc=newArray("1x1x1","4x4x4");

	Dialog.create("Setup");
	Dialog.addMessage("Random Removal of Signal   \n");
	Dialog.addMessage("- - - - - - - - - - - - - - -");
	Dialog.addChoice("Signal Removal up to: ", sval, sval[0]);
	Dialog.addChoice("Dimension of signal cluster: ", bloc, bloc[0]);
	Dialog.show();

	perc=Dialog.getChoice();
	Bdim=Dialog.getChoice();
	setBatchMode(true);

// Choice of the source Image stack
	showMessage("Please select the source image stack");
	open();
	tit=getTitle();

// Choice of the folder where results will be saved
	showMessage("Where do you prefer to save the resulting image?");
	dirT = getDirectory("Saving Folder");

// Let's get source image dimensions
	rawStack = getImageID();
	dimX = getWidth();
	dimY = getHeight();
	dimZ =nSlices()+1;

// Main cycle
	if(Bdim=="1x1x1") {
```

```
            if (perc=="5%") {
                  lim=0.05;
                  fix="5less";
            }
            else {
                  if (perc=="10%") {
                        lim=0.1;
                        fix="10less";
                  }
                  else {
                        lim=1;
                  }
            }

// Setup of target image
            resTit = fix+tit;
            newImage(resTit,"8-bit black",dimX,dimY,dimZ-1);
            run("Invert LUT");
            resStack = getImageID();

            if (lim<1) {
                  for (sl=1;sl<dimZ;sl++) {
                        showProgress(sl/dimZ);
                        random("seed",sl);
                        for(j=0;j<dimY;j++) {
                              for(i=0;i<dimX;i++) {
                                    selectImage(rawStack);
                                    setSlice(sl);
                                    val=getPixel(i,j);
                                    if(val==255) {
                                          rnd=random;
                                          if (rnd>lim) {
                                                selectImage(resStack);
                                                setSlice(sl);
                                                setPixel(i,j,255);
                                          }
                                          else {
                                          }
                                    }
                              }
                        }
                  }
            }
      }
      else {
            if(Bdim=="4x4x4") {
                  if(perc=="5%") {
                        lim=0.05;
                        fix="5less";
                  }
                  else {
                        if(perc=="10%") {
                              lim=0.1;
                              fix="10less";
                        }
                        else {
                              lim=1;
                        }
                  }

// Setting up mask
                  workTit = fix+"Mask";
                  resTit = fix+tit;
```

```
                    newImage(workTit,"8-bit black",dimX,dimY,dimZ-1);
                    run("Invert LUT");
                    workStack = getImageID();
                    makeRectangle(0, 0, dimX, dimY);
                    setForegroundColor(0, 0, 0);
                    run("Fill", "stack");

// Main depletion cycle for 4x4x4 clusters
                    if (lim<1) {
                          setForegroundColor(255, 255, 255);
                          for(sl=1;sl<dimZ/4;sl++) {
                                ice=((sl-1)*4)+1;
                                showProgress(ice/dimZ);
                                random("seed",sl);
                                for(j=0;j<dimY/4;j++) {
                                      k=j*4;
                                      for(i=0;i<dimX/4;i++) {
                                            l=i*4;
                                            setSlice(ice);
                                            rnd=random;
                                            if(rnd<lim) {
                                                  selectImage(workStack);
                                                  run("Specify...",     "width=4
height=4 x="+l+" y="+k+" slice="+ice);
                                                  run("Fill", "slice");
                                            }
                                      }
                                }
                          }
                          for (sl=1;sl<dimZ/4;sl++) {
                                ice=((sl-1)*4)+1;
                                run("Specify...","width="+dimX+"   height="+dimY+"
x=0 y=0 slice="+ice);
                                run("Copy");
                                for(w=1;w<4;w++) {
                                      ice++;
                                      setSlice(ice);
                                      run("Paste");
                                }
                          }

// Filling trailing slices
                          selectImage(workStack);
                          lastSlice=floor(dimZ/4)*4;
                          for(es=lastSlice+1;es<dimZ;es++) {
                                setSlice(es);
                                makeRectangle(0, 0, dimX, dimY);
                                setForegroundColor(0, 0, 0);
                                run("Fill", "slice");
                          }

// Intersecting original signal stack and randomly depleted mask
                          imageCalculator("AND create stack", rawStack,workStack);
                          resStack = getImageID();
                          selectImage(workStack);
                          run("Select None");
                          saveAs("tiff",dirT+workTit);
                          close();
                    }
              }
        }

// Closing the source image
```

```
        selectImage(rawStack);
        run("Select None");
        close();
// Saving and closing the result image
        selectImage(resStack);
        run("Select None");
        saveAs("tiff",dirT+resTit);
        close();
// End of macro operations
        setBatchMode(false);
        showMessage("End of Elaboration");
}
```

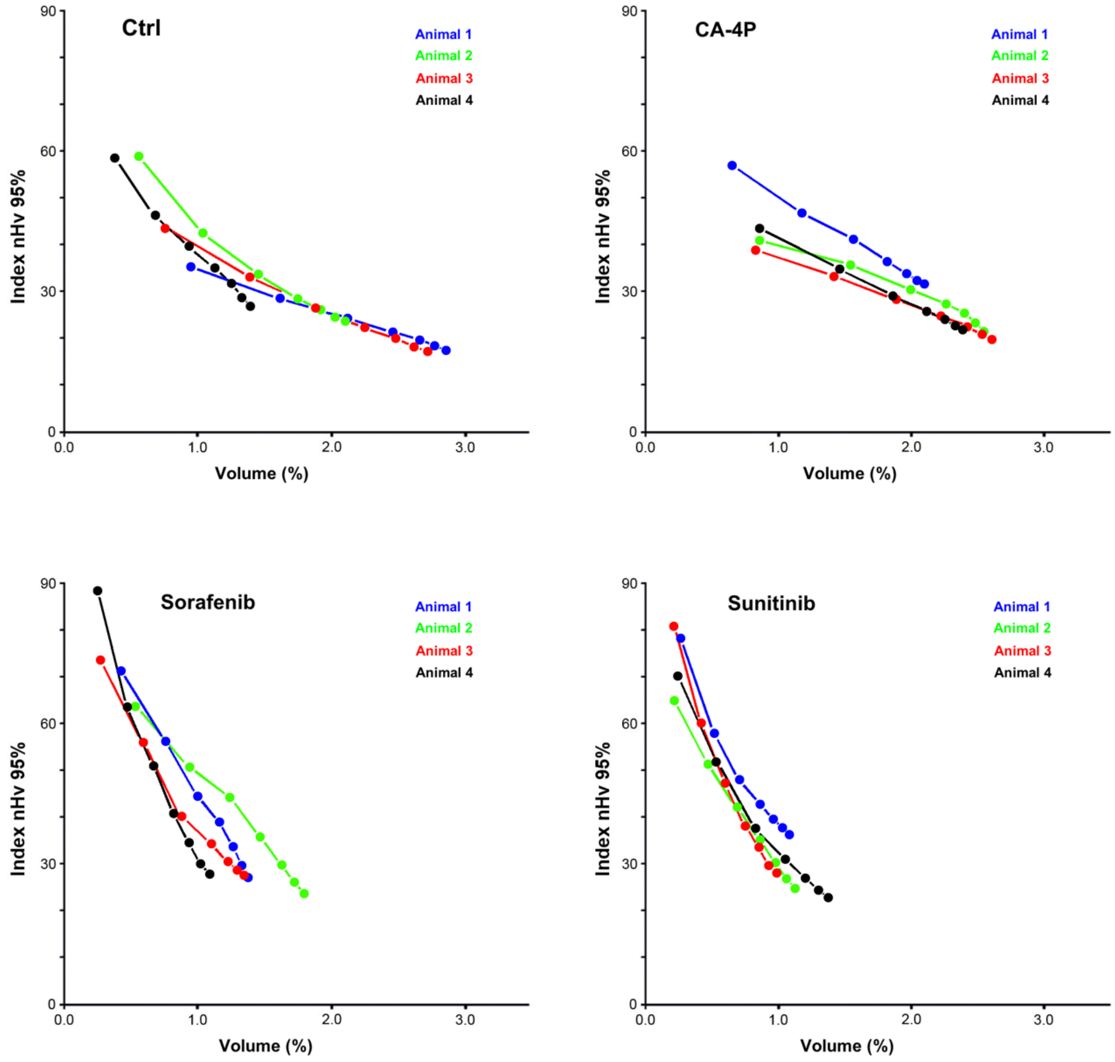

Figure S1. Tumor micro-angioarchitecture: inter-animal variability for different pharmacological treatments.

Median curves were obtained from the analysis of four tumors for each group of treatment (two z-stack per tumor; one tumor per animal). Each animal is represented by a different color.

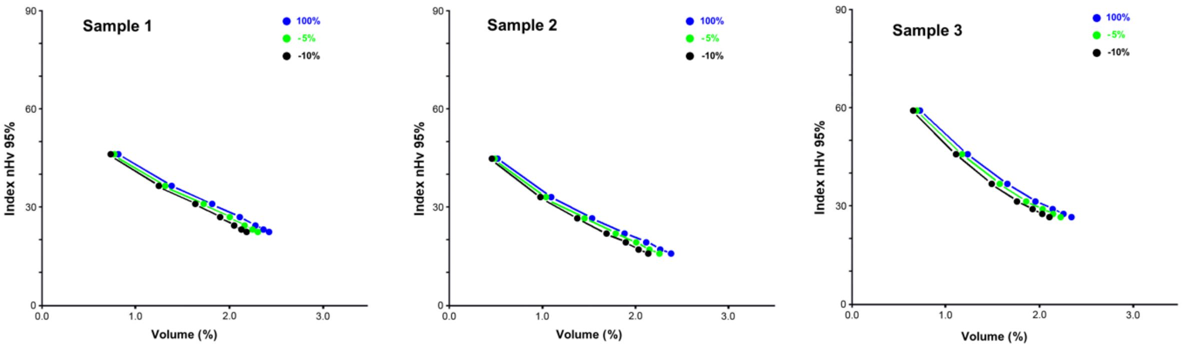

Figure S2. Robustness of the 3D microvascular analysis with respect to signal loss and vessel disconnection.

Three tumors grown in three different animal were analyzed. Each panel shows the curves obtained from a single z-stack before (blue curves) or after randomly depletion of 5% (green curves) or 10% (black curves) of the vascular signal by a custom routine (see Script S2). We randomly removed vascular signal from the binary volumes following two different approaches. In the first case, we randomly removed voxels, independently one from the other. In the second case, we divided the original volume into 4x4x4 cubes and removed all the signal voxels from randomly selected 64-voxel subvolumes. This last approach was aimed to mimic loss of areas of faint signal in order to obtain a further vessel disconnection. The depleted volumes were then intersected with sample-specific, caliber-classified masks to get the new vascular arrangements that were then combined into new series of partially reconstituted trees and analyzed for signal amount and dispersion. Only curves obtained by the first approach were reported in the figure since differences between the two approaches were not appreciable and curves were superimposable.

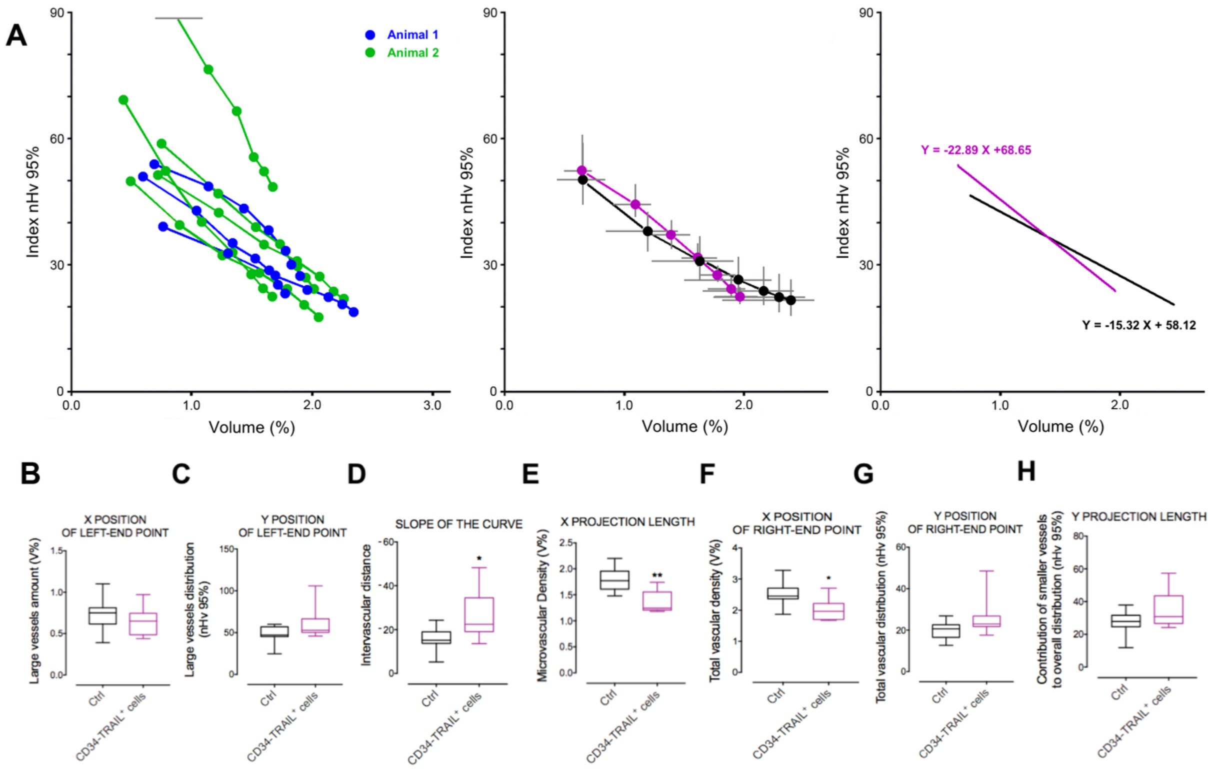

Figure S3. Analysis of tumor micro-angioarchitecture after treatment with CD34–TRAIL+ cells.

A) *Left panel:* Curves obtained from the analysis of 8 tumor z-stacks obtained from 2 different CD34–TRAIL+ cells-treated animals. Z-stacks belonging to the same animal are highlighted with the same color. Out of graph points are indicated by a gray line. *Middle panel:* Median curves from control (black) and CD34-TRAIL+ cell-treated (purple) samples. Median values (dots) and IQR (gray lines) for both percent volume and spatial dispersion are shown for each class of vascular trees. *Right panel:* linear regression curves calculated from median curves shown in the middle panel. **B-H)** Box and whiskers plots of curve-derived vascular parameters. The boxes extend from the 25th to the 75th percentiles, the lines indicate the median values, and the whiskers indicate the range of values.

* $p<0.05$; ** $p<0.01$.

KMS-11 tumors	Ctrl-1	Ctrl-2	Ctrl-3	Ctrl-4	Ctrl-5	Ctrl-6	Ctrl-7	Ctrl-8
**R^2**	0.998	0.995	0.984	0.955	0.990	0.994	0.979	0.993
**Slope (ℝ)**	-14.52	-5.23	-19.94	-24.29	-15.34	-11.28	-20.87	-42.63
**X/Y coords of left-end point**	0.82/46.38	1.10/24.58	0.73/59.87	0.39/58.87	0.59/45.02	0.92/42.51	0.46/54.03	0.30/63.52
**X/Y coords of right-end point**	2.42/22.64	3.28/12.68	2.34/26.85	1.86/21.00	2.43/16.02	3.01/18.73	1.65/27.57	1.13/26.58
**X length**	1.61	2.20	1.61	1.48	1.85	2.08	1.19	0.84
**Y length**	23.74	11.90	32.50	37.87	29.00	23.77	26.46	36.93

Table S1. Values of the descriptive parameters obtained from the curves of control KMS-11 tumors.

Vascular amounts and dispersion data from each point of cumulated vascular arrangements were used for each individual sample to obtain an interpolating line by linear fitting. Fitted lines were characterized in terms of R^2, slope (ℝ), X/Y coordinates of the first (left-end point) and last (right-end point) points and the X/Y length after projection on the respective axes. N = 4 mice; the two z-stacks belonging to the same animal are highlighted by the same color code as in Figure S1.

KMS-11 tumors	Soraf-1	Soraf-2	Soraf-3	Soraf-4	Soraf-5	Soraf-6	Soraf-7	Soraf-8
R^2	0.988	0.975	0.997	0.982	0.957	0.985	0.973	0.940
Slope (ℝ)	-57.63	-35.20	-27.34	-36.81	-37.51	-48.86	-38.80	-101.70
X/Y coords of left-end point	0.37/83.49	0.48/59.72	0.58/61.99	0.51/66.03	0.31/73.22	0.23/74.82	0.29/68.13	0.21/109.3
X/Y coords of right-end point	1.24/31.16	1.51/23.76	1.97/22.57	1.62/25.37	1.51/26.05	1.18/29.74	1.16/32.29	1.02/24.05
X length	0.87	1.03	1.41	1.12	1.21	0.94	0.86	0.81
Y length	52.33	35.96	39.42	40.66	47.17	45.07	35.84	85.29

Table S2. Values of the descriptive parameters obtained from the curves of Sorafenib-treated KMS-11 tumors.

Vascular amounts and dispersion data from each point of cumulated vascular arrangements were used for each individual sample to obtain an interpolating line by linear fitting. Fitted lines were characterized in terms of R^2, slope (ℝ), X/Y coordinates of the first (left-end point) and last (right-end point) points and the X/Y length after projection on the respective axes. N = 4 mice; the two z-stacks belonging to the same animal are highlighted by the same color code as in Figure S1.

KMS-11 tumors	Sunit-1	Sunit-2	Sunit-3	Sunit-4	Sunit-5	Sunit-6	Sunit-7	Sunit-8
R^2	0.982	0.943	0.994	0.974	0.968	0.974	0.946	0.988
Slope (ℝ)	-35.32	-75.73	-54.57	-37.71	-60.75	-72.46	-49.60	-32.63
X/Y coords of left-end point	0.30/65.59	0.22/91.67	0.24/66.29	0.19/64.29	0.25/75.80	0.17/86.48	0.21/80.43	0.27/60.65
X/Y coords of right-end point	1.34/27.80	0.80/45.53	0.91/30.32	1.33/19.99	0.95/31.24	1.01/25.60	1.28/24.86	1.45/21.51
X length	1.04	0.58	0.66	1.14	0.69	0.85	1.07	1.18
Y length	37.80	46.14	35.96	44.30	44.56	60.88	55.58	39.14

Table S3. Values of the descriptive parameters obtained from the curves of Sunitinib-treated KMS-11 tumors.

Vascular amounts and dispersion data from each point of cumulated vascular arrangements were used for each individual sample to obtain an interpolating line by linear fitting. Fitted lines were characterized in terms of R^2, slope (ℝ), X/Y coordinates of the first (left-end point) and last (right-end point) points and the X/Y length after projection on the respective axes. N = 4 mice; the two z-stacks belonging to the same animal are highlighted by the same color code as in Figure S1.

KMS-11 tumors	CA-4P-1	CA-4P-2	CA-4P-3	CA-4P-4	CA-4P-5	CA-4P-6	CA-4P-7	CA-4P-8
R^2	0.999	0.988	0.963	0.972	0.991	0.997	0.992	0.993
Slope (ℝ)	-21.54	-12.92	-15.12	-8.28	-18.06	-6.33	-26.72	-5.20
X/Y coords of left-end point	0.71/61.98	0.60/52.47	0.79/47.65	0.93/34.55	0.61/49.91	1.04/28.29	0.48/59.53	1.24/27.80
X/Y coords of right-end point	2.25/29.03	1.93/34.67	2.27/24.77	2.80/18.46	1.95/25.92	3.24/13.92	1.71/25.80	3.04/18.35
X length	1.54	1.33	1.47	1.87	1.34	2.19	1.23	1.80
Y length	32.96	17.14	22.87	16.09	23.99	14.37	33.73	9.45

Table S4. Values of the descriptive parameters obtained from the curves of CA-4P-treated KMS-11 tumors.

Vascular amounts and dispersion data from each point of cumulated vascular arrangements were used for each individual sample to obtain an interpolating line by linear fitting. Fitted lines were characterized in terms of R^2, slope (ℝ), X/Y coordinates of the first (left-end point) and last (right-end point) points and the X/Y length after projection on the respective axes. N = 4 mice; the two z-stacks belonging to the same animal are highlighted by the same color code as in Figure S1.

Data points (μm^2)	V (%)		nHv 95%	
Ctrls	Median	IQR	Median	IQR
**37 - 75**	0.66	0.40	50.20	14.59
**19 - 75**	1.20	0.60	38.07	9.32
**9 - 75**	1.63	0.68	31.08	10.22
**5 - 75**	1.95	0.72	26.46	9.27
**2 - 75**	2.16	0.76	23.98	9.21
**1 - 75**	2.29	0.76	22.55	9.10
**0 - 75**	2.38	0.77	21.82	8.60
CA4P	Median	IQR	Median	IQR
**37 - 75**	0.75	0.35	48.78	21.25
**19 - 75**	1.35	0.61	42.61	16.45
**9 - 75**	1.75	0.78	35.91	12.83
**5 - 75**	1.99	0.87	31.53	10.14
**2 - 75**	2.12	0.88	28.91	9.54
**1 - 75**	2.20	0.89	26.94	8.40
**0 - 75**	2.26	0.91	25.29	8.26
Sorafenib	V (%)		nHv 95%	
**37 - 75**	0.34	0.21	70.68	11.97
**19 - 75**	0.67	0.31	52.92	11.17
**9 - 75**	0.95	0.33	44.16	7.80
**5 - 75**	1.15	0.33	38.65	5.19
**2 - 75**	1.27	0.35	32.40	3.93
**1 - 75**	1.33	0.36	27.56	4.85
**0 - 75**	1.38	0.37	25.71	6.12
Sunitinib	V (%)		nHv 95%	
**37 - 75**	0.23	0.05	71.05	16.68
**19 - 75**	0.46	0.06	55.97	10.06
**9 - 75**	0.69	0.18	44.18	9.96
**5 - 75**	0.86	0.26	36.72	8.07
**2 - 75**	0.99	0.30	31.65	8.70
**1 - 75**	1.08	0.36	28.60	7.45
**0 - 75**	1.15	0.40	26.70	6.53

Table S5. Variability of single data points for different pharmacological treatments. The median V (%) and nHv 95% values (together with their relative IQR) are shown for each class of partially reconstituted vascular trees (data points).

KMS-11 tumors	Ctrls	CD34-TRAIL+ cells
R^2	0.999	0.994
Slope (ℝ)	-15.32 (-15.78/-14.85)	-22.89 (-24.89/-20.88)
X/Y coords of left-end point	0.75 (0.13) / 46.91 (7.40)	0.65 (0.24) / 52.66 (10.65)
X/Y coords of right-end point	2.45 (0.20) / 20.66 (5.00)	1.96 (0.36) / 22.98 (3.29)
X length	1.77 (0.27)	1.25 (0.33)
Y length	27.83 (3.61)	30.88 (10.24)

Table S6. Quantification of micro-vascular changes after CD34-TRAIL$^+$ cells treatment. Data are from the analysis of eight 3D vascular samples (z-stacks) obtained from two KMS-11 tumor grafts per group of treatment. For each type of treatment, median vascular amounts and dispersion data from each point of cumulated vascular arrangements were used to obtain an interpolating line by linear fitting. Fitted lines were characterized in terms of R^2, slope (ℝ), X/Y coordinates of the first (left-end point) and the final (right-end) points and the X/Y length after projection on the respective axes together with their relative IQR inside brackets.
